# Social vulnerability, impacts and adaptations strategies in the face of natural hazards: insight from riverine islands of Bangladesh

**DOI:** 10.1186/s12889-023-16497-8

**Published:** 2023-09-06

**Authors:** Babul Hossain, Guoqing Shi, Chen Ajiang, Md. Salman Sohel, Liu Yijun

**Affiliations:** 1 Management Science and Engineering, Hohai University, Nanjing, 210000 China; 2 National Research Center for Resettlement, Hohai University, Nanjing, 210000 China; 3 Research Center for Environment and Society, Hohai University, Nanjing, 210000 China; 4 Department of Development Studies, Daffodil International University, Dhaka, 1216 Bangladesh; 5 School of Public Administration, Hohai University, Nanjing, 211000 China

**Keywords:** Natural hazards, Preparedness, Public health, Social vulnerability, Disaster management, Emergency management

## Abstract

**Background:**

Bangladesh is one of the countries at risk of natural disasters due to climate change. In particular, inhabitants of its riverine islands (char) confront ongoing climatic events that heighten their vulnerability. This study aims to assess social vulnerability, impacts, and adaptation strategies to climate change in the riverine island areas of Bangladesh.

**Methods:**

A mixed-method approach incorporating qualitative and quantitative procedures was used on data collected from 180 households of riverine islands in Gaibandha, Bangladesh. The social vulnerability of riverine island communities was assessed based on their adaptation capacity, sensitivity, and exposure to climatic stressors.

**Results:**

The findings show that char dwellers' vulnerability, impacts, and adaptation capability to climate change vary significantly depending on their proximity to the mainland. Social vulnerability factors such as geographical location, fragile and low-grade housing conditions, illiteracy and displacement, climate-sensitive occupation and low-income level, and so on caused to the in-height vulnerability level of these particular areas. This study also displays that climate change and its associated hazards cause severe life and livelihood concerns for almost all households. In this case, the riverine dwellers employed several adaptation strategies to enhance their way of life to the disaster brought on changing climate. However, low education facilities, deficiency of useful information on climate change, poor infrastructure, and shortage of money are still the supreme hindrance to the sustainability of adaptation.

**Conclusion:**

The findings underscore the importance of evaluating the susceptibility of local areas to climate change and emphasize the need for tailored local initiatives and policies to reduce vulnerability and enhance adaptability in communities residing in char households.

## Introduction

In the twenty-first century, climate change has emerged as a prominent global issue, presenting numerous difficulties and challenges [1]. Its adverse effects pose a significant threat to the international community, particularly to developing nations that are highly susceptible to the severity, regularity, and intensity of natural disasters and severe weather events [2–4]. Climate change manifests in various forms, including changes in temperature, precipitation patterns, growing sea levels, and indications of severe weather [5, 6]. Furthermore, its far-reaching consequences profoundly impact global economic, social, and political activities, causing disruptions to people's ways of life [7, 8]. Extensive literature supports the notion that developing countries, including Bangladesh, are particularly exposed to the impacts of climate change [2, 9].

Bangladesh, with its distinctive geographical location, precarious socio-economic situations, increasing populace, widespread poverty, and limited technological infrastructure, stands as a nation highly susceptible to climate change [8]. Statistical projections predict a rise in sea levels by nearly 25 cm and 1 m by 2050 and 2100, respectively, leading to the displacement of 33 million and 43 million individuals during those periods [10, 11]. Additionally, Bangladesh is witnessing a steady increase in average temperature, with projections indicating a rise of 1.0 °C and 1.4 °C by 2030 and 2050, respectively, primarily attributed to the effects of climate change [2]. The country ranks fifth in vulnerability to severe weather incidents globally [12]. As a result, Bangladesh experiences a range of intense climatic events, including floods, riverbank erosion, cyclones, waterlogging, salinity intrusion, landslides, storm surges, and droughts on an annual basis [13, 14]. These events pose substantial encounters to the way of lives of its inhabitants [15].

This study focuses the vulnerability of the riverine island of Bangladesh, known as Char, to the impacts of natural hazards brought on by climate change. Char land, formed over 2–3 years by sediment deposition and erosion, is largely disconnected from the mainland and historically marginalized [16]. This area is highly susceptible to disasters such as floods, riverbank erosion, storms, and droughts [17]. Around 4–5% of Bangladesh's population, most of whom are involved in agriculture, live on these riverine islands [18]. Char residents, especially those on islands, are extremely vulnerable to climate change, with their homes, farms, and crops at risk, potentially leading to increased poverty and food insecurity [19]. Limited access to essential resources exacerbates their risk of impoverishment. Frequent displacement due to climate change-related events like floods and droughts is also common among Char inhabitants [20].

Several studies have been conducted on climate change impacts, vulnerability, and adaptation in Bangladesh, primarily focusing on biophysical and environmental aspects with limited attention given to the national and social levels [21–29]. Notably, few have explored the social ramifications of climate change, especially at household, rural community, and district levels. This gap suggests a need for deeper investigation into the social determinants of vulnerability to climate-related disasters, the socio-economic factors driving individual responses, and the management of localized impacts. Understanding these local-level dynamics is key to designing tailored strategies rather than resorting to broad, one-size-fits-all solutions based on national-scale assessments. The complex interactions among societal, political, and ecological features that influence climate sensitivity, impact severity, and the range of coping and adaptation strategies are also crucial. Recognizing the importance of social vulnerability and acknowledging indigenous adaptation methods of local communities, as emphasized by previous research and the Intergovernmental Panel on Climate Change, enhances community resilience to climate change [2, 30]. Despite the breadth of literature on climate change in Bangladesh [31–37], research on the social vulnerability of riverine island communities in the context of climate change is scarce. Therefore, this study aims to address the following research questions: (a) What is the current level of social vulnerability among riverine islanders in Bangladesh? (b) What are the adaptation strategies employed by riverine islanders to mitigate their vulnerability in the face of climate change and its associated hazards? Consequently, this study employs a bottom-up assessment framework technique, as recommended by the United Nations Framework Convention on Climate Change (UNFCC), to evaluate social vulnerability to climate change in two Upazilas. In addition, this study assesses the ramifications of climate change and delineates context-specific strategies for adaptation. Ultimately, this study presents instances of how policymakers, development strategists, and other vested parties can adequately address the requirements of marginalized groups residing in rural areas of Bangladesh by formulating and executing climate change policies and initiatives.

## Materials and methods

### Study area and location: a synopsis

**Fig. 1:**
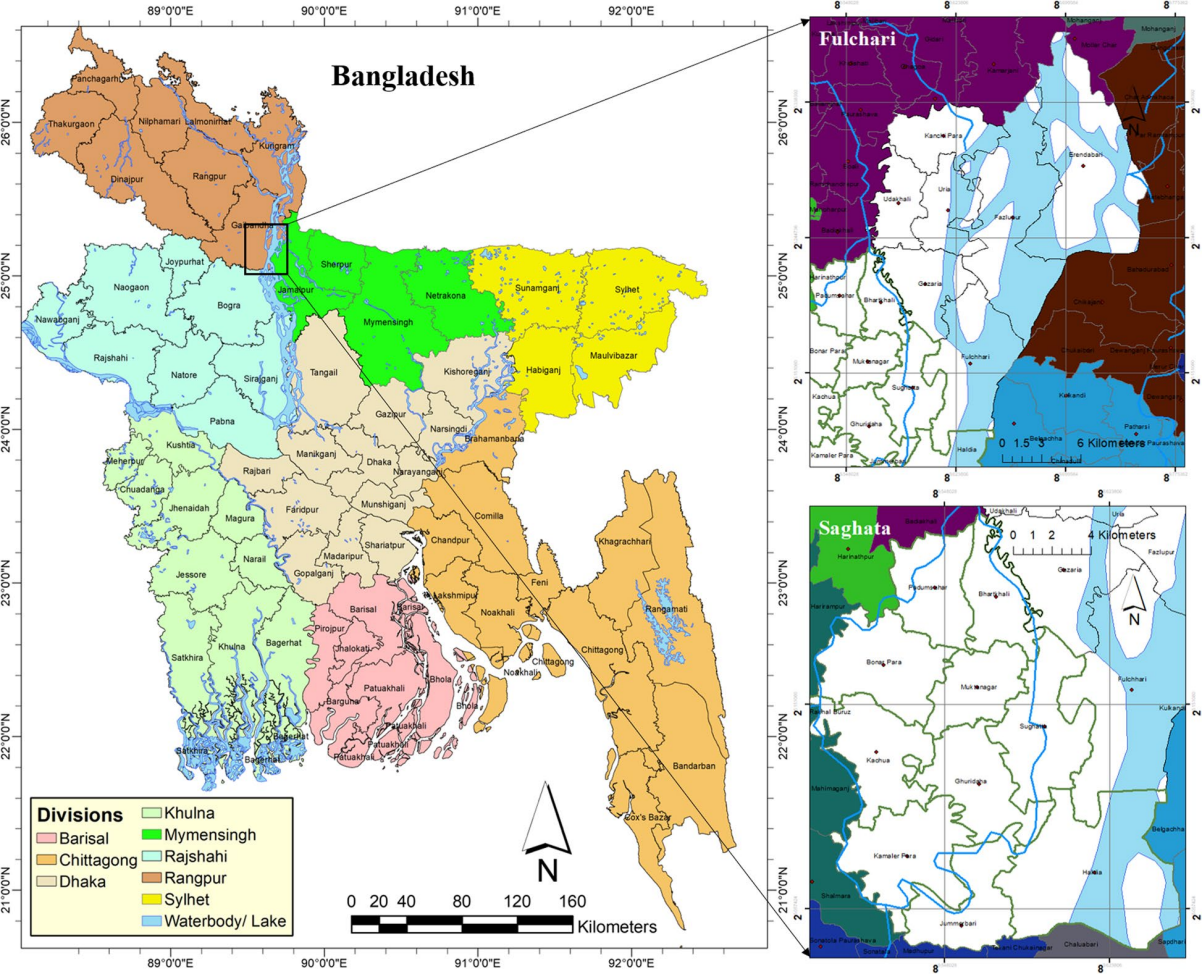
Location map of study area

Two Upazilas (sub-districts) from the Gaibandha district, namely Fulchari and Saghata, were chosen for the study (refer to Fig. 1). The investigation primarily focused on two distinct categories of riverine island areas: firstly, char residents residing within a 6 km range of Saghata Upazila, who are in close proximity to the mainland; and secondly, char residents residing more than 6 km away from the mainland, specifically from the Fulchari Upazila headquarters. The same level of climate change-induced hazards frequently affects each of these places. However, each has a distinct character in terms of the Upazila’s communication system, the district's administrative center, educational and medical facilities, as well as other essential public services and resources for subsistence. The studied villages in Fulchari Upazila were Kalur Para and Bajefulchari, and Sathalia and Haldia were from Saghata Upazila, respectively.

### Sampling, and data collection

This study employed a mixed-methods strategy, integrating both qualitative and quantitative methods. In this study, a purposive sampling method was utilized to choose households for interviews and the distribution of questionnaires. The criterion for choosing households within the study villages was the presence of a household head (HH) aged 30 years or older. A total of 45 houses per village voluntarily agreed to participate in the interviews and questionnaire distribution, resulting in a total sample size of 180 households. A pilot survey was conducted on 20 household to assess the suitability of the data collection instrument. The findings from the pilot survey were used to develop the data collection instrument. Field surveys were then carried out using semi-structured questionnaires, field visits, literature review, and expert opinions. Quantitative data were gathered from a questionnaire survey of 180 households while qualitative measures were employed for data triangulation, and information was got together through FGDs, depth interviews, KIIs, and observations. The FGDs included 6–12 participants and 2–3 research team members, focusing on essential issues. KIIs were conducted with local leaders and individuals from government and non-government organizations, addressing concerns about disasters and climate change's impact on char areas' inhabitants. The survey was carried out from January to March 2022.

### Measuring approach of social vulnerability to climate change

**Fig. 2:**
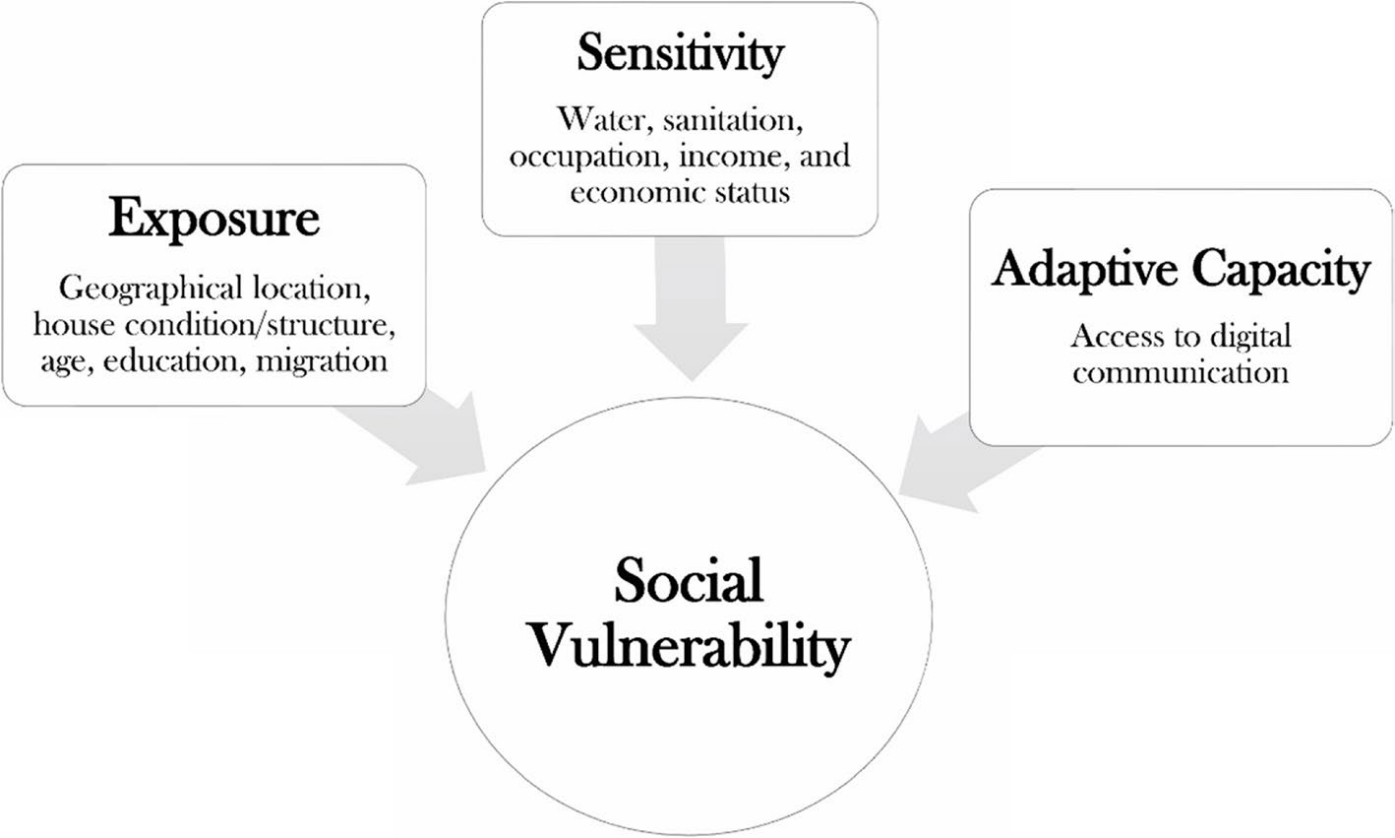
Indicators in this study for social vulnerability assessment

This study evaluated the social vulnerability of Bangladesh's riverine island areas using the bottom-up assessment framework technique suggested by the United Nations Framework Convention on Climate Change [38]. Social vulnerability to climate change is understood as a function of exposure, sensitivity, and adaptive capacity [39]. Therefore, this study assessed some of the indicators to measure the social vulnerability like exposure (geographical location, house condition, age, education, and migration), sensitivity (water, sanitation, occupation, income or economic status), and adaptive capacity (access to digital communications) under the three components (Fig. 2). This strategy has been employed in numerous climate studies and research papers, including those from The United States Agency for International Development [40], The International Union for Conservation of Nature (IUCN) [41, 42], and Turner et al*.*, (2003) [43].

### Measurement of climate change impacts and adaptation strategies

This research focused on understanding the impact of climate change on riverine island inhabitants by scrutinizing five livelihood assets: human capital, social capital, financial capital, physical capital, and natural capital. The study considered various factors related to these assets to assess the extent of climate change effects. A three-tiered classification system was used to examine the impact of changing climate on the way of life of people living on Char lands, categorized as low, medium, and high scales. Additionally, 26 variables were used to examine the adaptation strategies employed by residents of Char dwellings to mitigate the consequences of climate change. The study also classified households into three categories (low popular, medium popular, and high popular) based on their observations of the actual scenarios faced by island inhabitants due to changing climate (as outlined in Table 2). The adaptation techniques were divided into two categories: individual-level adaptation (ILA) and planned adaptation (PA), which were commonly used by riverine island populations [17].

### Statistical analysis

After gathering various data, the obtained data were scrutinized according to the research objectives. Predominantly, this research focused on descriptive analysis where quantitative data underwent analysis utilizing statistical tools such as the Statistical Package for Social Sciences (SPSS). Conversely, manually the qualitative information was accentuated through textual and document analyses. Tables, charts, and graphs were concurrently arranged into distinct categories to enhance the applicability and comprehensibility of the content for the intended readership. Furthermore, social vulnerability indicators were generated by utilizing primary data obtained from a survey of 180 households. Moreover, the examiners provided their interpretations based on the findings and observations derived from the assessment of both primary and secondary data, as well as from interviews conducted with the informants. Charts and graphs were used represent the major dimension of social vulnerability.

## Results

The findings of this study have been illustrated in several subsections. The first two subsections detail the household vulnerability and impacts of the riverine islander in the face of climate change and its associated hazards and disasters. The subsequent subsections elucidate the adaptation strategies employed by the inhabitants, alongside a discussion of the associated barriers and challenges.

### Influencing factors of social vulnerability to climate change

**Fig. 3:**
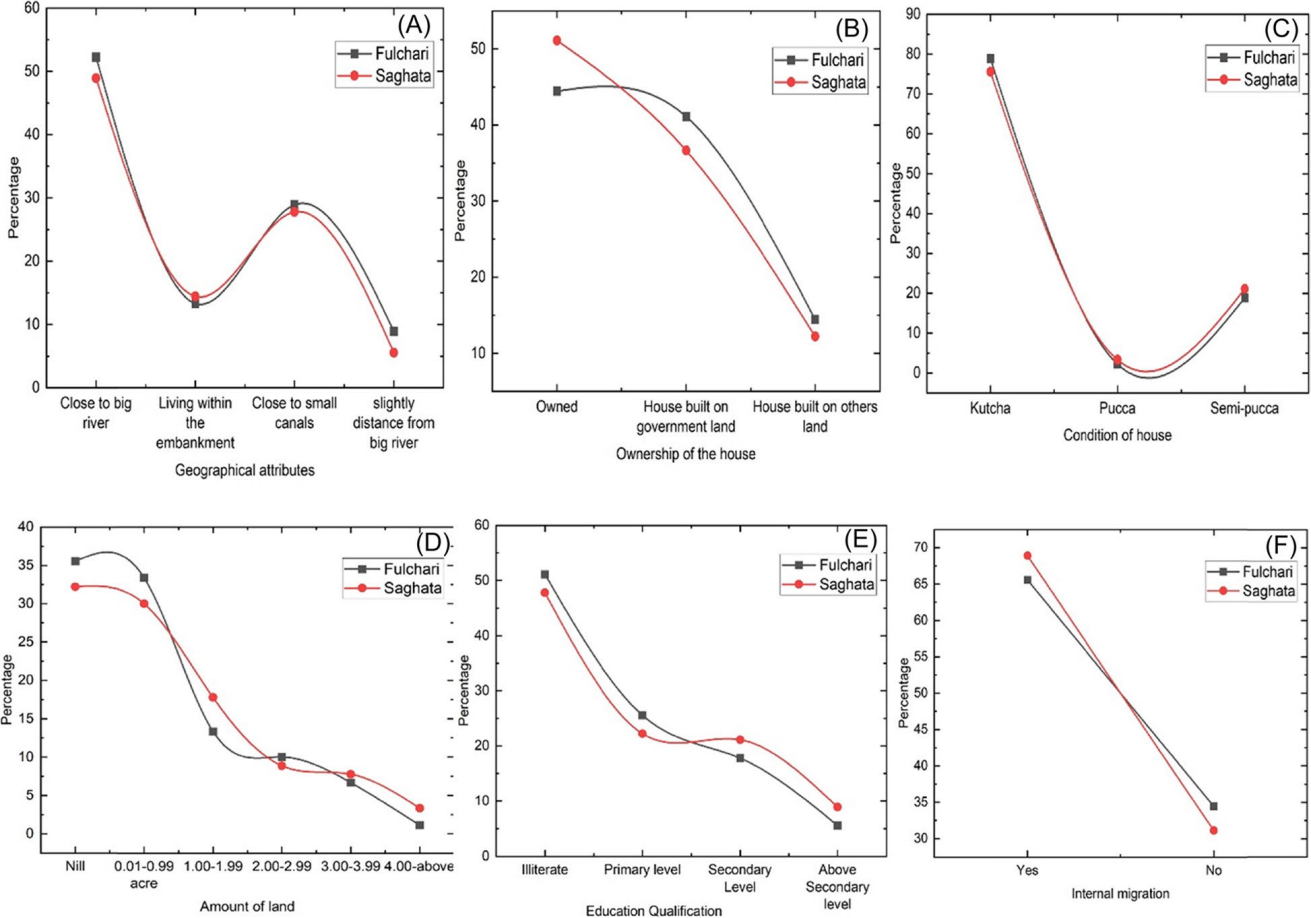
Key components of exposure for social vulnerability analysis, Source: field survey

The following Fig. 3 displays the key elements of exposure to analyze the study area's social vulnerability. The geographical location of the study area unfolded that almost half of the participants in two sub-districts (52.22% from Fulchari and 48.89% from Saghata Upazila) live close to the main Jamuna River, and some char inhabitants reside a little distance from the main river in the study area. Besides, approximately 28.89% and 27.78% of riverine island dwellers dwell near the tiny canals in Fulchari and Saghata Upazila. Moreover, the rest of the participants live within the embankment (Fig. 3A). Therefore, based on geographical location, the people dwelling in Fulchari Upazila are more defenseless than those dwelling in Saghata Upazila.

In the designated study region, majority of respondents' dwellings (78.89% in Fulchari and 75.56% in Saghata Upazila) were categorized as kutcha, indicating houses made of mud and straw. A smaller proportion (18.89% in Fulchari and 21.11% in Saghata Upazila) were semi-pucca houses, while only a minority were classified as pucca, constructed with materials like bricks, cement, and stone. Focused Group Discussions revealed that many individuals in the study area, including jhupri houses made of cottage materials, mud, and weak wood, reside in kutcha houses with pillars (Fig. 3C). More than half of the respondents' residences are located on government or privately-owned lands, while 45.45% in Fulchari and 51.11% in Saghata Upazila have built their houses on their own land (Fig. 3B). Overall, the availability of housing facilities appears to be more favorable in Saghata Upazila compared to Fulchari Upazila. A respondent stated his/her sorrows concerning the housing facilities as follows**-**

*“We are poor men. We have no own land to cultivate, even don’t have housing land. So, we live in a tent made of plastic and bamboo on government land, including children and disabled family members. Also, we have limited social services opportunities such as pure drinking water, sanitation, and children's education facilities. So, we don't even know how our life will go; on top of that, every year, floods destroy everything. At that time, we have nothing to do to survive against the disasters with particular family members” (Interviewee, F-27).*

Figure 3 (D) shows that a significant portion of respondents, approximately 35.56% and 32.22% respectively, had no agricultural land at all. In terms of non-agricultural land, which mainly includes residential areas, the majority of respondents owned only a small amount of farmland. Less than 1.11% and 3.33% of participants possessed more than four acres of land in the surveyed villages. These findings highlight a higher prevalence of landless individuals in Fulchari compared to Saghata Upazila, emphasizing the relatively lower land ownership in Fulchari.

In terms of education, a considerable proportion of the surveyed individuals, specifically 51.11% in Fulchari and 47.78% in Saghata, lacked basic literacy skills and were unable to read or write. Additionally, a significant number of respondents had completed primary and secondary education. However, only a small percentage of participants, specifically 5.56% and 8.89%, pursued education beyond the secondary level, as shown in Fig. 3E. Concerning education, a key informant stated his/her opinion that “*most children between 5 to 14 years old come to school regularly; after that, they do not attend school because of poverty. Besides, the ratio of girls is not satisfactory to go to school. As a result, they are not qualified to know about the means the life and responsibility properly. However, the educational facilities are not well here” (KII # 5).* In contrast, the riverine island zones experience ongoing internal migration caused by various social and economic factors. Figure 3 (F) provides empirical evidence showing that a considerable portion of the population has relocated from their original homes at least once in their lives.

**Fig. 4:**
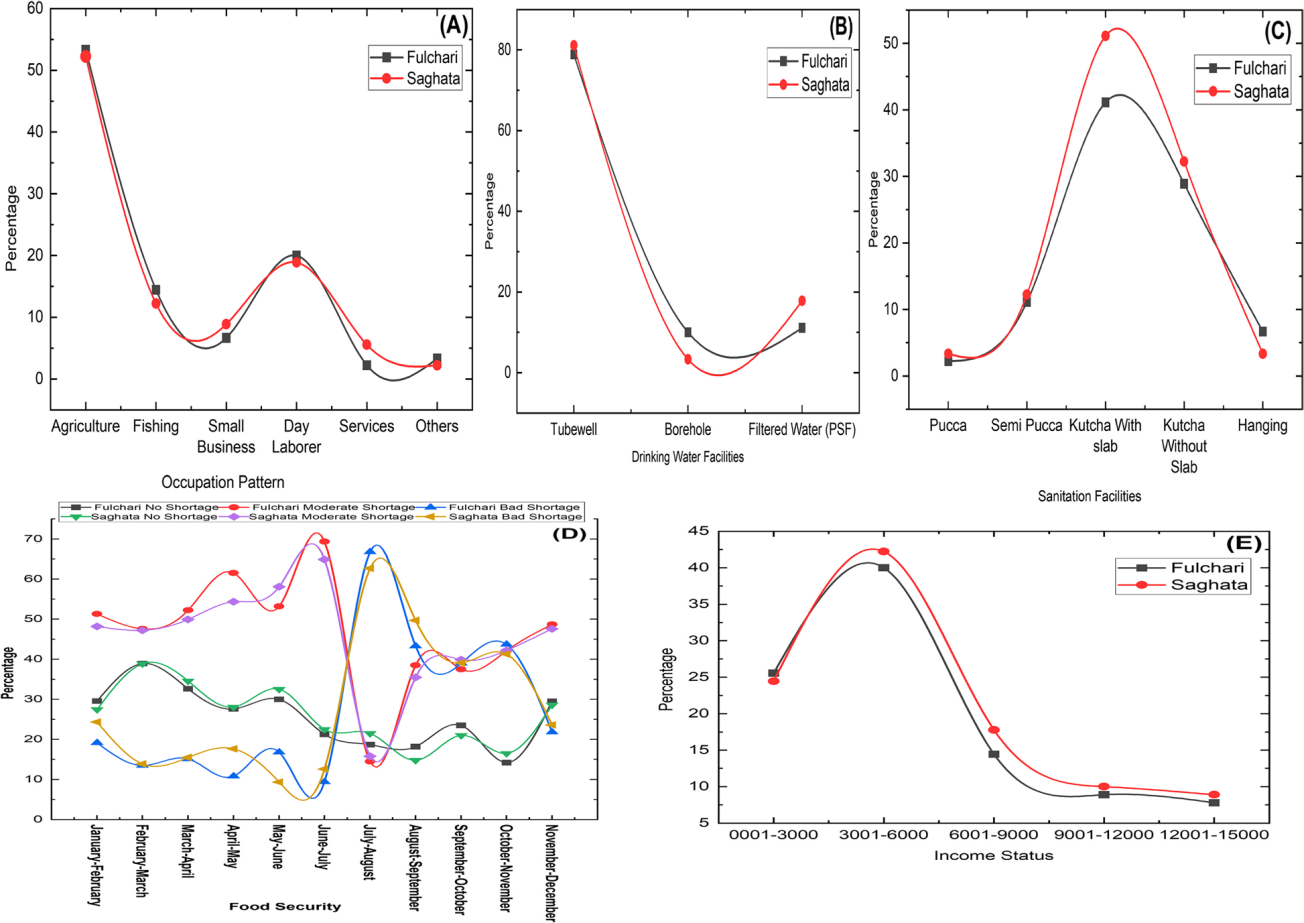
Significant components of sensitivity for social vulnerability analysis, Source: field survey

Figure 4 (A) show that agriculture and day labor are the dominant occupations in both Fulchari and Saghata sub-districts, with a higher prevalence in Fulchari. The income distribution analysis reveals that a significant number of respondents in both areas earn between 3001 to 6000 Bangladeshi Taka, while a considerable percentage have incomes ranging from 1 to 3000 Taka (Fig. 4E). Food shortages are observed throughout the year, particularly between July and November (Fig. 4D). These findings highlight the continuous internal migration and social vulnerability experienced by the inhabitants of riverine islands in Fulchari and Saghata. Regarding water sources, a significant proportion of respondents in Fulchari and Saghata (approximately 81.11% and 78.89% respectively) rely on tube wells as their primary source of drinking water (Fig. 4B). Some respondents also use filtered water (PSF) and nearby boreholes. However, the sanitation facilities in the study area are comparatively fragile, with a substantial number of households (41.11% in Fulchari and 51.11% in Saghata) using kutcha toilets with a slab. Kutcha toilets without a slab and hanging latrines are also utilized by some households. It is important to note that the availability of pucca and semi-pucca sanitation facilities was limited within the study area, as depicted in Fig. 4 (C).

**Fig. 5:**
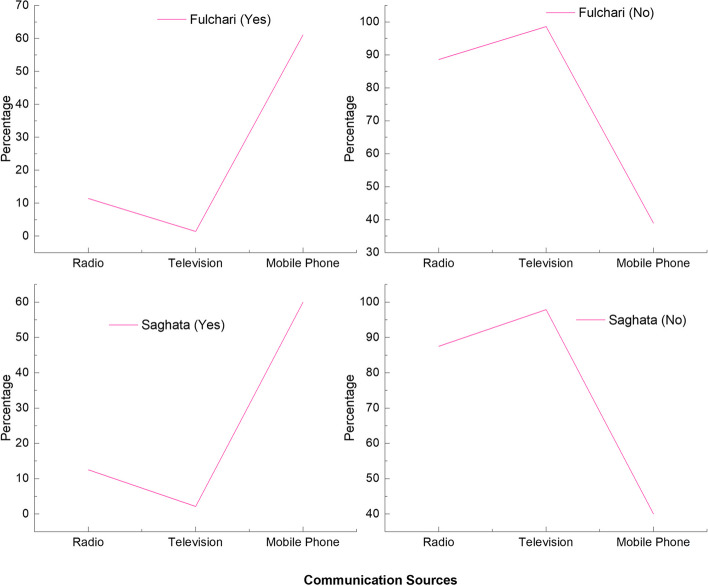
Communications sources in the study villages, Source: field survey

Despite the well communication practices observed among rural inhabitants, the expeditious and efficient dissemination of preparedness and response information can be achieved through the employment of radio and television mediums. Nonetheless, this investigation has divulged that merely a limited number of households possess radios or televisions, whereas certain respondents possess mobile phones, which assume pivotal significance in times of crisis. Furthermore, a notable proportion of individuals inhabiting "kutcha homes" exhibit a dearth of access to televisions and radios, thereby potentially reflecting their economic standing and purchasing capacity relative to their counterparts (see Fig. 5).

### Climate change impacts (CCI) on the riverine island households

The findings revealed that residents faced significant challenges related to flooding, erosion, and other climate-related risks. These challenges affected various aspects of their lives and livelihoods.

Table 1 showed that both Fulchari and Saghata Upazilas experienced considerable difficulties due to climate change. In terms of human capital, both areas faced food insecurity, health issues, and educational obstacles, with Saghata being more severely affected. Social capital also suffered, with deteriorating social bonds and household healthcare services in both areas, though Saghata had slightly higher percentages of challenges. Financial capital challenges included the lack of loan facilities, employment and income source obstacles, insufficient savings, and crop loss. Physical capital challenges encompassed housing infrastructure, sanitary facilities, asset loss, energy services, transportation disruptions, and embankment damage. Fulchari was more vulnerable in terms of physical capital. Overall, the study highlighted the extensive impact of climate change and related disasters on the villages, with both Upazilas facing similar challenges. The revelations from KIIs and FGDs unveiled that the substantial implications of climate change observed in the study area primarily stem from alterations in crucial climatic factors.

*“I am such an unlucky person that I could not protect my property because I am now getting old. What else I say about my sorrow? I had a cow; it looked like a tiger. People said hey, older! this is the time to sell, so do not be late. But I planned to sell out this in the winter when my daughter's marriage will be set up. Hi, my fate; what else I say? All have been ruined. Now I have nothing else to bear the cost of my daughter's marriage, oh Allah! I cannot think anymore; why did you keep me here?” (Interviewee # F-37)*

**Table 1:** The views of people living in riverine islands regarding the effects of climate change

Sorts of assets	CCI	Study Location					
		**Fulchari (%)**			**Saghata (%)**		
		Low	Medium	High	Low	Medium	High
Human capital	Food uncertainty and starvation	6.67	35.56	57.77	18.89	35.56	45.55
	Health complications	3.33	63.33	33.34	8.89	58.89	32.22
	Education	27.78	45.56	26.66	26.67	52.22	21.11
	Job loss	23.33	61.11	15.56	22.22	65.56	12.22
	Migration	24.44	64.44	11.11	20.00	66.67	13.33
Social capital	Social connection	35.55	57.78	6.67	32.22	62.22	5.56
	Religious foundations	65.56	31.11	3.33	63.33	32.22	4.45
	Health services	28.89	31.11	40.00	26.67	53.33	20.00
Financial capital	Loan facilities	37.78	40.00	22.22	34.44	44.45	21.11
	Work and income	18.89	57.78	23.33	23.33	53.33	23.34
	Savings	3.33	21.11	75.56	7.78	27.78	64.44
	Yields	-	18.89	81.11	-	30.00	70.00
Physical capital	Housing	10.00	46.67	43.33	15.56	51.11	33.33
	Hygiene assistance	15.56	40.00	44.44	23.33	41.11	35.56
	Farming assets	30.00	57.78	12.22	27.78	61.11	11.11
	Non-agricultural equipment’s	23.33	63.34	13.33	21.11	67.78	11.11
	Electricity (Solar/DB)	12.22	54.44	33.34	16.67	56.67	26.66
	Transport	-	46.67	53.33	-	56.67	43.33
	Embankment	35.56	52.22	12.22	40.00	52.22	7.78
Natural capital	Land	32.22	41.11	26.67	30.00	45.56	24.44
	Drinking water	-	25.56	74.44	-	28.89	71.11
	Livestock	21.11	56.67	22.22	23.33	61.11	15.56
	Fisheries	13.33	35.56	51.11	10.00	40.00	50.00
	Social forestry	40.00	46.67	13.33	35.56	44.44	20.00
	Soils	14.44	40.00	45.56	18.89	46.67	34.44

Source: Field survey

The older man incurred the loss of his sole possession as a result of a torrential water current. Even though his family had no income sources because his sons separated from him, now he is living with his older wife and daughter. It is noteworthy that, he has been suffering from mental problems since the last flood.

### Adaptation mechanism of char households to climate change

The riverine inhabitants dwelling in char regions encounter significant challenges arising from climate change and the resultant disasters. In light of this situation, the vulnerable residents typically devise multiple adaptation strategies aimed at bolstering their resilience to climate change. These strategies predominantly rely on individual-level adaptations stemming from experiential and acquired knowledge, alongside planned adaptations that receive backing from governmental and non-governmental organizations. The adaptation strategies employed by the riverine island dwellers to enhance climate change resilience are outlined in Table 2.

The research findings presented in Table 2 illustrate the responses of residents in Fulchari to the effects of climate change and its connected catastrophes. The findings reveal that the majority of households in Fulchari adopted measures aimed at reducing expenditure, decreasing food consumption and storage, conserving water resources, diversifying crops, utilizing organic fertilizer, planting trees, and implementing homestead gardening. These strategies were universally embraced as the most prevalent means to alleviate the negative consequences of climate change. However, FGDs disclosed that the local population, facing economic disadvantages, implemented somewhat planned procedures to address climate change impacts, focusing on reducing food consumption, stockpiling provisions, and engaging in livestock rearing. Additionally, other adaptation strategies of medium and low popularity were identified, including seasonal migration, petty business ventures, off-farm employment, and highland plantation initiatives. In contrast, respondents from Saghata utilized similar strategies, such as reducing food consumption and storage, decreasing expenditure, diversifying crops, and implementing homestead gardening, to mitigate climate change impacts. They also implemented various other adaptation plans based on their capabilities. It is worth noting that respondents predominantly sought medical treatment from government hospitals and unqualified practitioners due to the lack of healthcare facilities in the area.

**Table 2:** Adaptation tactics of the household in the riverine island’s villages

Adaptive measure	Responses (%)		Tactics popularity		Remarks
	Fulchari	Saghata	Fulchari	Saghata	
Reduce food consumption and food storage	85.56	82.22	xxx	xxx	PA/ILA
Lessen expenditure	94.44	92.22	xxx	xxx	PA/ILA
Seasonal Migration	61.11	52.22	xx	xx	ILA
Conservation of water resources	70.00	74.44	xxx	xxx	ILA/PA
Crop diversification	78.89	81.11	xxx	xxx	ILA
Use of organic fertilizer	71.11	65.56	xxx	xx	ILA/PA
Tree plantation	86.67	76.67	xxx	xxx	
Homestead gardening	74.44	71.11	xxx	xxx	ILA/PA
Change in planting and harvesting time	67.78	73.33	xx	xx	ILA/PA
Conversion of agricultural land	12.22	11.11	x	x	ILA
Floating garden	10.00	6.67	x	x	ILA/PA
Livestock rearing	76.67	83.33	xxx	xxx	ILA
Plantation in highlands	46.67	43.33	xx	xx	ILA/PA
Wave protection walls	17.78	14.44	x	x	PA
Grow leafy vegetables to cover walls and roofs	35.56	41.11	xx	xx	ILA
Cage Aquaculture/ Net aquaculture	30.00	28.88	x	x	ILA/PA
Construction of embankments	40.00	35.56	xx	xx	PA
Re-digging of canal	48.89	45.56	xx	xx	PA
Reduced tillage and deep plowing	63.33	71.11	xx	xx	PA
Take treatment (Govt. hospital, Quack doctor)	100.00	100.00	xxx	xxx	ILA/PA
Housing components facilities (GOs and NGOs)	15.56	17.78	x	x	PA
Taking loan (NGOs, moneylender, relatives and neighbor)	92.22	96.67	xxx	xxx	ILA/PA
Off farm employment (Van, rickshaw, nachimon, korimon and tempo, driver)	52.22	54.44	xx	xx	ILA
Petty business	32.22	25.56	x	x	ILA/PA
Frequent consultation with extension officers	28.89	34.44	x	x	PA
Training on CC	21.11	25.56	x	x	PA

Source: Field survey; Several answers were considered

xxx = high popular, xx = medium popular, x = low popular; *ILA* Individual level adaptation based on experience and knowledge, *PA* Planned adaptation (supported by GOs & NGOs)

The outcomes from FGDs revealed that monetary resources are regarded as the foremost essential prerequisite for effectively addressing the harmful penalties of changing climate. In this context, the practice of rearing goats, chickens, and ducks, as well as engaging in cage/net aquaculture, emerged as a prompt means of attaining financial stability among the study respondents, with approximately 76.67% and 83.33% of the respondents reported engaging in livestock rearing in the Fulchari and Saghata Upazilas, respectively.

### Factors barrier the households’ adaptation strategies

Since the riverine island dwellers evolved a number of adaptation techniques to avoid dire circumstances, they also detected a variety of impediments to the adaptation strategies (Table 3).

**Table 3:** Components of adaptation barriers to climate change resilience

Obstacles	Level of seriousness			Rank
	Less Serious	Moderately serious	Very serious	
Low level of education	13.33	35.56	51.11	4
Deficient information on climate change	24.44	52.22	23.33	8
Low level of technology	-	57.78	42.22	5
Use of the traditional farming system	21.11	58.89	20.00	9
Lack of assets access	23.33	35.56	41.11	6
Paucity of money	-	21.11	78.89	2
Poor infrastructure	-	41.11	58.89	3
Lack of proper government support	-	16.67	83.33	1
Absence of the proper sense of responsibility to coordinate or act on adaptation practices	34.44	36.67	28.89	7

Source: Field survey; Multiple responses were considered. Rank has been calculated based on very serious scale

According to the findings presented in Table 3, individuals residing in the char regions exhibited a notable dearth in educational attainment and a lack of comprehensive knowledge concerning the subject of climate change. A significant portion of the participants regarded these factors as moderately serious and very serious impediments, corresponding to respective rankings of 4 and 8. To illustrate, one respondent expressed their viewpoint in the following manner:

*“We belong to a low-income family. My father always said that we don't have money to buy straws for the house and food, so how is it possible to send my children to school for education? It's not just ridiculous; it's nothing but luxury. But now, I understand how important education is to become a successful person in our life. For example, I am a day laborer; of course, I don't have the qualifications to get a good job, and I don't have a proper understanding of the government and other institutions' dissemination of climate change and its consequences. Because of my ignorance, my family faces several issues every year. My limited income and knowledge do not allow me to save money, including planning for the next catastrophe. Thus, our lives are becoming more fragile and vulnerable every day, making us hopeless” (Interviewee # S-14).*

The majority of the participants held the view that inadequate technological advancements and inadequate infrastructure constituted significant impediments to the process of acclimatization in the face of complications arising from climate change. Furthermore, the findings presented in Table 3 indicate that none of the respondents expressed their opinion on the less severe obstacles pertaining to adaptation strategies, assigning them ranks of 3 and 5, respectively. Conversely, a substantial proportion of riverine island residents, specifically 41.11% and 78.89%, regarded limited access to resources and financial scarcity as highly formidable barriers to adapting to climate change, assigning them ranks of 6 and 2, respectively. The focus group discussions (FGDs) revealed that the majority respondents in the study lacked sufficient economic resilience to navigate the challenges experienced in their lives and livelihoods. In relation to this issue, a study participant articulated their perspective in the following manner:

*“We are a group of homeless people residing in the demesne (Khas land). We lack the assets and cash needed to deal with the difficulties. We frequently sought financial assistance from many sources, including Mahajan, local NGOs, relatives, etc. However, most of the time, we are unable to fulfill their financial requirements. Although some of the people (Mahajan) agreed to lend us some money, the interest rate was quite high because we are not affluent enough to repay the loan with such a high-interest rate. As a result, we have to overcome many obstacles to address the robust issues with life and livelihood brought on by natural disasters” (Interviewee, F-19).*

Nevertheless, the utilization of conventional agricultural techniques and efficient synchronization were additional impediments to the process of adaptation. In summary, a proportion of 16.67% and 83.33% of the survey participants expressed their perception that insufficient government assistance posed a moderate and highly significant obstruction in deal with changing climate and its repercussions. Notably, no respondents indicated a perception of its triviality. Through FGDs and KIIs, it was uncovered that the general populace is deprived of governmental support due to inadequate coordination, corruption, nepotism, and other related factors. Nonetheless, the government does offer numerous provisions in response to the overwhelming circumstances induced by climate change and its related natural calamities.

## Discussion

The findings demonstrated minimal variation in Upazila’s under study regarding vulnerability. However, a thorough examination showed significant disparities between these two Upazilas in several instances regarding vulnerability. According to existing literature, measuring vulnerability includes identifying risks and considering resilience and regaining from the antagonistic consequences of climate change [44].

Following the social vulnerability measurement results, particularly, in the case of exposure, the geographical location of the survey area showed that approximately all people live close to the main river, either very close or slight distance and some lives in the embankment, which is also closer to the river. However, communities near rivers, embankments, or the sea are more vulnerable to floods, cyclones, storms, sea level rise, and even tsunamis [45]. Therefore, the geographical features of the communities provide insight into potential risks to the natural resources on which these people rely. In addition, the study identified three main types of houses in the area, all of which demonstrate the vulnerability of the entire community to natural disasters. Kutcha houses, in particular, are easily demolished and damaged during such events, as observed in other studies in different region [20, 46]. Furthermore, nearly half of the houses are situated on government or others' land, which reflects the social and financial conditions of the individuals in the community. Moreover, the construction materials used in temporary houses, such as bamboo and plastic, make them fragile and susceptible to climatic events like floods, erosion, and cyclones, increasing the vulnerability of the community. Many individuals in the study area had no agricultural land and very little land overall, indicating a poor socio-economic condition in the villages. The research shows that people living in poverty are particularly at risk and affected by natural disasters. Around half of the participants in the study area were illiterate, which is consistent with similar research conducted in other regions of Bangladesh [27]. As a result, households in the research area were not aware of modern adaptation measures and continued to rely on conventional practices, making them more vulnerable to climate change due to low literacy levels and insufficient extension services. The study also found that people migrated internally to different places due to frequent adversity in the area, and the departure of male family members made women socially and financially vulnerable. Migration was influenced by financial, social, and livelihood factors, as demonstrated in other research studies [47, 48].

In the context of sensitivity, agriculture, day labor, and fishing play a significant role in the livelihoods of households in the study areas, which heavily rely on natural resources. Researchers have identified the vulnerability of these livelihoods to climate change impacts in various locations [49, 50]. Furthermore, data reveals that many households lack alternative sources of income, making them more susceptible to the adverse effects of climate change. Poor and marginalized groups, such as day laborers, farmers, and fishers, are particularly at risk due to their dependence on environmental resources and limited options for income diversification [26, 51]. Insufficient monthly income resulting from climate-sensitive occupations further exacerbates their inability to meet basic needs like food, healthcare, and savings. Consequently, residents of char areas are highly exposed and vulnerable to climatic events. Additionally, the prevalence of inadequate housing structures, including kutcha with slab and kutcha latrine without slab, increases the risk of infectious diseases during the rainy season [17, 52, 53]. Drinking water is primarily sourced from tube wells, boreholes, and PSF, which have been identified as potential factors contributing to water- and vector-borne illnesses, such as diarrhea and influenza, during extreme flood disasters. These health issues disproportionately affect millions of low-income individuals in Bangladesh annually.

The study revealed regarding adaptive capacity that communication components like radio and television are not very satisfactory due to their economic hardship, which can signify the community's financial well-being and purchasing power. The vulnerability may increase as it becomes more difficult for local or state governments to properly disseminate information owing to a lack of radio and television. Even though social nets are widespread within communities, this knowledge is urgently needed in times of calamity. Thus, it was found that char dwellers' means of subsistence were highly vulnerable in all of the research sites. The results indicate that both populations of islanders are defenseless, but those closest to the mainland are less vulnerable than those far away. This is probably because public and non-profit groups offer facilities, there are more robust social and communication networks, and educational facilities. It is simple to move after significant calamities.

Therefore, it has ample evidence that the amplified frequency and strength of disasters and climate change significantly impact Bangladesh's riverine island dwellers. These climate-induced adversities, ubiquitously confronted by households, pose substantial challenges to their sustenance and lifestyle, notably impacting the agricultural sector upon which they heavily depend. The Char regions, in particular, report climate change ramifications such as food scarcity, unemployment, and educational disruptions, corroborating findings from Alam et al*.*, (2020) [54]. Additionally, the study identifies a correlation between these climate-related adversities and health afflictions, notably waterborne and vector-borne diseases, aligning with prior research [17, 55]. The research also underscores the societal consequences of internal migration, such as weakened familial and social ties, thereby increasing vulnerability due to the loss of social capital, which is compatible with the results of the study led by [56, 57]. In sum, participants' experiences echo the multifaceted impact of climate change, causing not only physical and economic distress but also significant societal and environmental concerns, mirroring broader research trends in the field [24, 48].

However, the char dwellers employed several adaption measures regarding their ability to manage their household income in the face of challenging circumstances. Likewise, Alam et al. (2017) revealed that various adaptation strategies taken by the char people, developed based on the level of each individual, or planned adaptation backed by GOs and NGOs, might be valuable to make sure the viable livelihood against the effects of climate change [24]. The riverine island acclimatized numerous strategies (Table 2) to climate change resilience. Considerable research has continuously emphasized that implementing advanced adaptation strategies promotes agricultural production and reduces poverty [58]. Households who want to reduce climatic perils and have sufficient gate to valuable sources are more adaptable and resilient [59]. As well as, figuring out the challenges to acclimatization in the face of climate change is crucial for creating prosperous livelihood coping strategies [60]. The most prominent element that limits the ability to adjust is the fluctuating financial situation among the numerous types of barriers [61, 62]. Similarly, one of the major obstacles for the riverine island people in the study area is the financial problem. Most of the responders engage in climate-sensitive occupations. In addition, participants in the study highlighted several barriers to addressing climate issues (Table 3); which is align with existing research in the field [63].

The outcomes of this research have practical implications for policymakers and governmental organizations. It suggests the need for cutting-edge adaptation plans to improve the adaptability, decrease vulnerability, and boost resilience of riverine island households. The study also contributes theoretically by formulating crucial indicators for evaluating social vulnerability, impact, and resilience among households in riverine islands. More precisely, the research findings have significant implications for national actions related to poverty reduction, improving conditions for the impoverished, livelihood projects on islands, and providing special assistance to socially excluded individuals. However, to address the socioeconomic vulnerabilities and hazards associated with disasters and climate change, efforts should be made to map and reduce extreme poverty in rural regions of Bangladesh, in line with the Sustainable Development Goals (SDGs). It is essential to develop catastrophe resilience manuals to classify an organization's systems, actions, and functioning conditions following disasters. Based on interviews, residents in char areas emphasize the importance of a comprehensive growth plan, including road infrastructure, sustainable forest management, year-round employment opportunities, and capacity building initiatives, to fortify their resilience against vulnerabilities. Enhancing communication quality, transportation facilities, and accessibility to essential public services is crucial to augment the adaptive capabilities of char dwellers.

## Conclusion

Bangladesh is particularly vulnerable to climate-induced natural calamities owing to its position. The vulnerability of char dwellers, regardless of location, is found to be associated with socio-economic factors. The study reveals that both island dwellers are susceptible to climate-induced natural disasters, with variations observed in terms of proximity to the mainland and different components and subcomponents. The main drivers of social vulnerability include climate-sensitive occupation, geographical location, lack of quality-of-life facilities, limited land, low access to food and non-food items, and financial hardship. Char dwellers face significant challenges in their daily lives and means of subsistence, rendering them more defenseless. Climate change and its associated hazards lead to housing and hygiene issues, food insecurity, health complications, job and crop loss, and countless other difficulties for the char land people. Although they have developed adaptation strategies such as reducing food consumption, crop diversification, tree plantation, livestock rearing, and consulting with extension officers, their livelihood resilience efforts are hindered by resource scarcity, low education levels, and financial constraints. The findings may be applicable to similar socio-economic perspectives in other riverine island areas or communities in different countries facing similar climate change challenges. This research is not free from limitations. Primarily, the data were collected from respondents based on their memory and perception. Additionally, the study only covered selected few variables. Further research could focus on exploring the effectiveness of specific adaptation strategies employed by riverine island communities in Bangladesh and their long-term sustainability, with more variables.

## Data Availability

The datasets supporting the findings of this article are available in this manuscript.
